# Willingness to Accept the COVID-19 Vaccine and Related Factors among Indian Adults: A Cross-Sectional Study

**DOI:** 10.3390/vaccines10071095

**Published:** 2022-07-08

**Authors:** Ashwaghosha Parthasarathi, Rahul Krishna Puvvada, Malavika Shankar, Jayaraj Biligere Siddaiah, Koustav Ganguly, Swapna Upadhyay, Padukudru Anand Mahesh

**Affiliations:** 1 Allergy, Asthma and Chest Centre, Krishnamurthypuram, Mysore 570004, India; ashwa.partha@gmail.com; 2 Department of Physiology, Anatomy, and Microbiology, College of Science Health and Engineering, La Trobe University, Melbourne 3086, Australia; 20173174@students.latrobe.edu.au; 3 Department of Internal Medicine, Interfaith Medical Center, New York, NY 11213, USA; mshankar@interfaithmedical.com; 4 Department of Pulmonology, JSS Medical College, JSS Academy of Higher Education & Research, Mysore 570015, India; bsjayaraj@jssuni.edu.in; 5 Unit of Integrative Toxicology, Institute of Environmental Medicine (IMM), Karolinska Institutet, 17177 Stockholm, Sweden; koustav.ganguly@ki.se

**Keywords:** COVID-19, health communication, COVID vaccine, public health, vaccine hesitancy, India, VAX scale

## Abstract

To achieve herd immunity to a disease, a large portion of the population needs to be vaccinated, which is possible only when there is broad acceptance of the vaccine within the community. Thus, policymakers need to understand how the general public will perceive the vaccine. This study focused on the degree of COVID-19 vaccine hesitancy and refusal and explored sociodemographic correlations that influence vaccine hesitancy and refusal. A cross-sectional online survey was conducted among the adult population of India. The survey consisted of basic demographic questions and questions from the Vaccination Attitudes Examination (VAX) Scale. Multinomial logistical regression was used to identify correlates of vaccine hesitancy and refusal. Of the 1582 people in the study, 9% refused to become vaccinated and 30.8% were hesitant. We found that both hesitancy and refusal predictors were nearly identical (lower socioeconomic status, female gender, and older age groups), except for three groups (subjects aged 45–64 years, those with approximate income <10,000 INR/month, and those residing in rural households) that showed slightly higher odds of vaccine hesitancy than refusal. We need to address the underlying sociodemographic determinants and formulate public awareness programs to address specific subgroups that are at higher risk of rejecting the vaccine and convert those who are undecided or hesitant into those willing to accept the vaccine.

## 1. Introduction

The coronavirus disease caused by Severe Acute Respiratory Syndrome, or Coronavirus 2 (SARS-CoV-2), was first identified in December 2019 (Wuhan, China) [1]. The infection spread like wildfire and by March of 2021 was responsible for 122 M cases and 2.7 M deaths worldwide [2]. While some countries (e.g., Sweden) initially pursued herd immunity, a number of countries successfully introduced preventative measures until vaccines became available [3,4,5]. The only viable public health response to the pandemic is developing herd immunity within the general population, estimated at 67% [6], and the development and deployment of an effective vaccine is the only viable way to achieve this. India’s national drug regulatory authority provided emergency-use authorization for two vaccines, one of which was developed in the UK and funded by the UK government. Subsequently, the government collaborated with AstraZeneca to produce the vaccine and market it at cost. In India, AstraZenaca collaborated in mass manufacturing with the Serum Institute of India to produce COVISHIELD [7]. The second vaccine was developed in-house by the Indian biotech company Bharath Biotech (COVAXIN). India started its vaccination initiative on 16 January 2021 with COVISHIELD followed by COVAXIN. The USFDA has still not approved COVISHEILD and COVACCINE but has approved vaccines from PFIZER and JANSEN, which are manufactured in the USA and are considerably more expensive.

At the time of writing (April 2021), nearly 120 million people have received one dose and 1.1% of the population has been fully vaccinated with two doses [8]. The WHO estimates that three in five Indians need to be vaccinated to reach herd immunity and that the country will need 1.45 billion doses of vaccine by May 2022 [9]. The achievement of this target, however, is facing grave setbacks, as only about 56% of eligible people have come forward to receive the vaccine [10]. The process of COVID-19 vaccination has seen an alarming increase in the widespread mistrust of vaccine safety and efficacy. This lack of trust may be due to the fast-track development of these vaccines, making their potential side effects unknown in the public’s eyes [11,12,13]. In combination with this widespread pandemic, the unwillingness to become vaccinated may lead to unacceptably high morbidity and mortality rates.

Overall, India is witnessing a low population turnout, with vaccine wastage of 6.5% [14]. Studies in other countries such as the UK, USA, and France observed similar concerns about accepting the COVID-19 vaccine [11,15,16]. Unfortunately, no published data covering a large nationally representative sample (*n* ≥ 1000) are available on the extent of vaccine refusal or hesitancy in India. Thus, there is an urgent need to understand the current attitudes toward vaccines and the refusal or hesitancy to be vaccinated. The findings of such studies will help policymakers tailor a more compelling public health message to the general population to promote vaccination. Therefore, the present study focused on the extent of negative attitudes (refusal and hesitancy) toward the COVID-19 vaccine and the independent factors associated with vaccine refusal and hesitancy.

## 2. Materials and Methods

Due to the large dispersal of COVID-19 across India and related movement restrictions, conducting an online survey enabled us to understand people’s perceptions of the COVID-19 vaccine and determine the predictors for vaccine refusal and hesitancy with minimum risk to the researchers. This study was approved by the Institutional Ethics Committee of JSS Medical College, Mysuru (JSSMC/IEC/110621/17NCT/2021-22). All the participants were asked to complete an informed consent form for their participation and the publication of the data. Consent was provided by means of a tick box before beginning the survey.

To conduct the study, a web-based instrument (Google Forms) was used. The participants were invited to participate via a hyperlink that explained the objective of the study and guaranteed total anonymity. The survey ran for 45 days, and two reminder emails were sent at regular intervals. The invite also contained contact information for the primary investigator to answer survey-related queries. For increased clarity and reproducibility, the study design followed the Strengthening the Reporting of Observational studies in Epidemiology (STROBE) guidelines (Table S1).

The questions in the survey were categorized into three domains. The first domain consisted of sociodemographic questions, and the other two domains related to the perception of the vaccine and anxiety about the pandemic. The questions about the perception of the vaccine were derived from four subgroups as determined by the standardized Vaccination Attitudes Examination (VAX) Scale [17]. Simultaneously, coronavirus anxiety was measured using the standardized Coronavirus Anxiety Scale (CAS) [18].

### 2.1. Study Participants and Design

A minimum target sample size was calculated using the Raosoft sample size calculator with a 5% margin of error, a 95% confidence interval, and a 50% response distribution. The minimum recommended sample size was 377. All adults (above 18 years of age) who consented to participate in the survey were considered eligible for the study. The survey was disseminated randomly to people via social network advertisements and mailing lists across India. We received responses from different parts of India: North (*n* = 427), South (*n* = 538), West (*n* = 259), and East (*n* = 358). The survey included details such as the contact information of the principal investigator, the number of questions in each domain, and the approximate time to complete the survey. The survey was open for 45 days from mid-February to March 2021. The participants were assured of the confidentiality of the data provided. Participants who voluntarily participated in the survey and completed all the questions were included in this study. To avoid duplication of data, each participant was restricted to one response per email address.

### 2.2. Definitions and Measures

#### 2.2.1. Vaccine Intentions

A scale of 1 to 6 was used to gauge intention to take the vaccine. A score of 1–2 was considered as unlikely/refusal to accept the vaccine, 3–4 was considered hesitant, and 5–6 indicated a high likelihood to accept the vaccine.

#### 2.2.2. Vaccine Attitudes

Questions concerning vaccine attitude and intentions were assessed using a 12-item Vaccination Attitudes Examination (VAX) Scale [17], which was further subclassified into four predetermined [17] subgroups: (a) mistrust of vaccine benefits, (b) worries about unforeseen future effects, (c) concerns about commercial profiteering, and (d) preference for natural immunity. In these subclasses, a score of 5–6 out of a maximum of 6 was considered a high score, 3–4 was deemed to be intermediate, and 1–2 was considered a low score. The scores indicated the levels of negative attitudes toward the vaccine.

#### 2.2.3. Predictor Variables

Sociodemographic factors included gender (male vs. female), age group (70+, 50–69, 30–49, and 18–29), education, income, and dwelling area (urban or rural). Coronavirus anxiety during the previous two weeks was measured using the Coronavirus Anxiety Scale (CAS). The CAS contains five items assessing physical symptoms of anxiety. Responses are on a 5-point scale ranging from “not at all” to “nearly every day.” A CAS score of nine or more classified adults as meeting (90% sensitivity) or not meeting the (85% specificity) threshold for Generalized Anxiety Disorder [18]. We categorized responses such that participants with one or more COVID-19 anxiety symptoms were compared to those who did not report any such symptoms.

Responses to the question on compliance with government COVID-19 guidelines were assessed on a scale from 1 to 7, with 1 being “none at all” and 7 being “very much so.” Knowledge of COVID-19 was assessed using a six-question survey-based questionnaire developed by Zhong et al. [19], which was validated in previous studies [20,21]. Each of the six questions was given a single point, thus providing a scale ranging from 1 to 6, where a total score of 1 to 3 was considered poor knowledge and 4 to 6 was considered good knowledge.

### 2.3. Statistical Analysis

Data from the online survey forms were exported into Microsoft Excel 2017. EpiInfo™ 8.5.1 (2008) was used for calculating the frequencies. Missing data were addressed by using the case deletion approach wherein we omitted cases with missing data and analyzed the rest [22]. Continuous variables were represented as the mean with standard deviation (SD) and categorical variables as a number with percentage (%). A simple chi-square test was employed to test for differences in vaccine use among categorical variables.

Multivariable analyses using SPSS v.21 were conducted to identify independent variables associated with vaccine refusal and hesitancy. Variables presumed to be of importance, such as demographic factors (e.g., age, sex, and socioeconomic status) and interpersonal factors (such as knowledge of COVID-19) were included in the model. A two-tailed result of *p* < 0.05 was considered statistically significant.

## 3. Results

Of the 2,046 total participants who initially consented, 1582 participants completed all the questions in the survey and 464 did not complete the survey. Of these 1582 participants, more than half of the responses were received from males (59.7%) and more than two-thirds (68.7%) were urban residents. Approximately 40% of responses were received from participants in the age group 25–44 years, followed by 18–24 years (29.1%), 45–64 years (27.6%), and ≥65 years (3.9%). Almost 22% of the population had an average monthly income of 99,900–74,700 INR, followed by 17.5% earning 74,700–49,900 INR. More than 25% of the participants were not willing to disclose their income. In terms of education, 36.2% of responses were received from those with an undergraduate degree and 27.2% from those with a postgraduate degree. Participants who completed at least high school composed 18.4% of the sample. The majority of responses were received from participants living in urban areas of South India (34.6%), followed by North India (26.4%). The majority (76.5%) of participants had good knowledge of COVID-19. Around 61% of participants adhered to government COVID-19 guidelines. The majority of participants (64.3%) also declared that they were never diagnosed with COVID-19, and 87.3% of participants never had coronavirus anxiety symptoms (Table 1).

### 3.1. Perception of Vaccines

**Figure 1:**
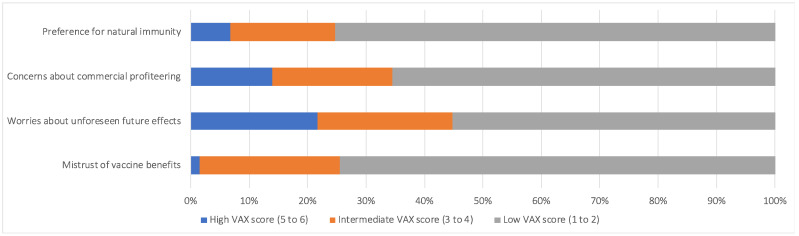
Levels of negative attitudes toward the vaccine. The study participants reported high, intermediate, and low VAX scores representing the levels of negative attitude toward the vaccine (*n* = 1582). The question about the perception of the vaccine was derived from the four subgroups determined by the Vaccination Attitudes Examination (VAX) Scale.

The majority of participants (74.5%) expressed low levels of mistrust in vaccine benefits. More than half of the participants (55.2%) were worried about unforeseen long-term adverse effects from the vaccine. Around 66% of the participants expressed concerns about commercial profiteering from vaccines, and 75.3% expressed willingness to develop natural immunity. Overall, from the results of our study most participants showed a low level of negative attitude toward the vaccines under all four domains (Figure 1).

**Figure 2:**
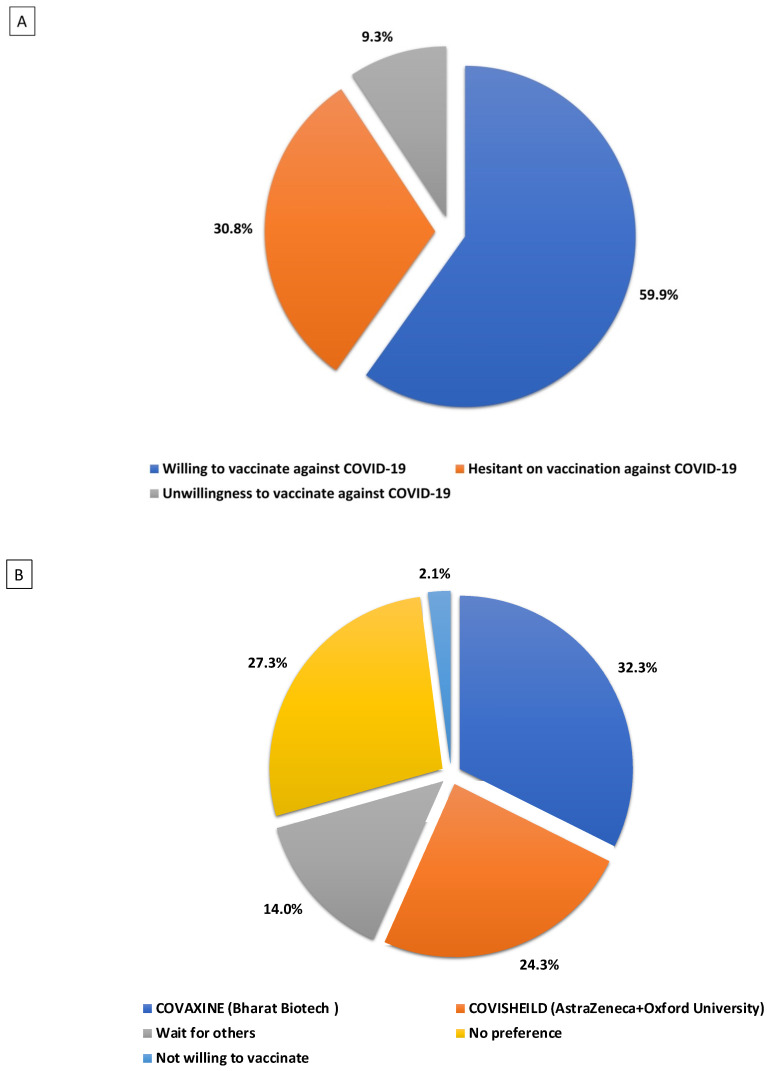
(**A**) Vaccine intentions. The proportion of vaccine intentions measured in the study population of *n* = 1582. Almost 60% were willing to vaccinate, 30% were hesitant about vaccination, and 10% were unwilling to vaccinate. (**B**) Type of vaccine preferred in the study population. The type of vaccine against COVID-19 preferred in the study population of *n* = 1582. The majority of the population preferred either COVAXINE (32.3%) or COVISHEILD (24.3%).

The majority of the survey participants (60%) expressed willingness to take the COVID-19 vaccine, 30.8% were hesitant, and 9.3% refused to take the vaccine (Figure 2A).

### 3.2. Type of COVID-19 Vaccine Preferred

From the results of our study, more than half of the participants (56.7%) expressed that they would prefer to take either of the two vaccines marketed in India, i.e., COVISHIELD or COVAXIN. Around 24.3% of our study participants specified that they would prefer to take the COVISHIELD vaccine, which AstraZeneca is marketing in collaboration with the Serum Institute of India, and 32.3% of participants chose COVAXIN, which was developed by Bharat Biotech in collaboration with the Indian Council for Medical Research. Approximately 27% of the total participants expressed no preference for any particular brand of vaccine, and 14% of participants said they would wait for other vaccine manufacturers to market in India. Significantly, few participants (2.1%) expressed refusal to become vaccinated with any available vaccine brands (Figure 2B).

### 3.3. Predictors of Refusal to Vaccinate against COVID-19

**Figure 3:**
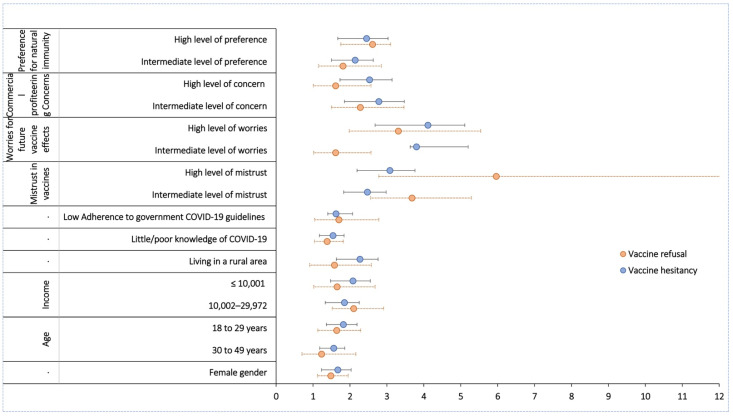
Predictors of COVID-19 vaccine refusal and hesitancy. Predictors of COVID-19 vaccine refusal and hesitancy using multivariable logistic regression analysis.

From the results of our study, we found that refusal to take the COVID-19 vaccine was higher among females (AOR = 1.18; 95% CI: 1.19-1.95), participants aged 18–29 years (AOR = 1.64; 95% CI: 1.13–2.29), those with poor knowledge of COVID-19 (AOR = 1.38; 95% CI: 1.04–1.82), participants with family income of 10,002–29,972 INR (AOR = 2.1; 95% CI: 1.52–2.9), and participants who were less adherent to government COVID-19 guidelines (AOR = 1.7; 95% CI: 1.04–2.78). Furthermore, refusal to take the vaccine was greater among participants who had high (AOR = 5.96; 95% CI: 2.78–12.77) or intermediate (AOR = 3.68; 95% CI: 2.56–5.29) mistrust of vaccine benefits, high (AOR = 3.31; 95% CI: 1.98–5.54) or intermediate (AOR = 1.61; 95% CI: 1.02–2.57) concerns about unforeseen future effects of vaccine, high (AOR = 1.61; 95% CI: 1.01–2.57) or intermediate (AOR = 2.28; 95% CI: 1.5–3.46) concerns about commercial profiteering, and high (AOR = 2.61; 95% CI: 1.75–3.9) or intermediate (AOR = 1.81; 95% CI: 1.15–2.84) preference for natural immunity (Figure 3).

### 3.4. Predictors of Hesitancy to Vaccinate against COVID-19

Apart from the identification of predictors for refusal to take the vaccine, we identified predictors for the participants’ hesitancy toward acceptance of the COVID-19 vaccine. We found that females (AOR = 1.67; 95% CI: 1.31–2.11) and participants 30–49 (AOR = 1.56; 95% CI: 1.26–1.94) and 18–29 years of age (AOR = 1.82; 95% CI: 1.45–2.28) were most hesitant to take the vaccine. Moreover, hesitancy to take the vaccine was observed in participants with family income of 10,002–29,972 INR per month (AOR = 1.85; 95% CI: 1.45–2.37) and ≤10,001 INR (AOR = 2.08; 95% CI: 1.61–2.69), those living in rural areas (AOR = 2.27; 95% CI: 1.78–2.91), those with poor knowledge of COVID-19 (AOR = 1.54; 95% CI: 1.24–1.91), and those who were less adherent to government COVID-19 guidelines (AOR = 1.69; 95% CI: 1.36–2.12). Furthermore, hesitancy to accept the COVID-19 vaccine was greater among participants with intermediate (AOR = 2.47; 95% CI: 1.96–3.11) or high (AOR = 3.08; 95% CI: 2.4–3.97) mistrust of vaccine benefits, intermediate (AOR = 3.8; 95% CI: 2.4–3.97) or high (AOR = 4.11; 95% CI: 3.11–5.54) concerns about unknown side effects of the vaccine, intermediate (AOR = 2.78; 95% CI: 2.09–3.71) or high (AOR = 2.53; 95% CI: 1.92–3.33) concerns about commercial marketing, and intermediate (AOR = 2.14; 95% CI: 1.65–2.77) or high (AOR = 2.45; 95% CI: 1.87–3.22) preference for natural immunity (Figure 3).

## 4. Discussion

The COVID-19 pandemic has resulted in unprecedented mortality and morbidity worldwide [2]. Vaccination, along with other health measures, is imperative to control the pandemic. This goal has been extremely challenging in the present time, as negativity toward the benefits of vaccines in general is increasing [23]. Global studies on the negative perception of vaccine use can help identify key indicators of such behavior [6,7,17], but very little is known about vaccine refusal and hesitancy among the general population of India.

Our study identified three sociodemographic factors associated with vaccine refusal (Figure 3). Females, those with lower levels of household income (<29,872 INR/month), and younger participants (18–29 years) were associated with an increased likelihood of vaccine refusal. Less adherence to government guidelines on COVID-19 prevention protocols was also strongly associated with a higher probability of vaccine refusal. Of all the predictors for rejection of the vaccine, mistrust of the benefits of the vaccine had the strongest association. We also identified predictors for vaccine hesitancy and found that the predictors for both hesitancy and refusal were nearly identical (Figure 3). This factor made it extremely difficult for us to distinguish between those subjects who will never take the vaccine and those who are hesitant but may accept the vaccine if they are convinced that it is beneficial. Future research should explore the differences between these two groups. Identifying these differentiating factors could help us understand the roots of vaccine hesitancy, and addressing such issues might help us convince those with vaccine hesitancy to accept the vaccine.

Consistent with previously published research findings from high-income countries, our study showed that subjects from lower socioeconomic brackets were two times more likely than others to refuse the vaccine [12,15,16]. Several studies from LMICs did not include socioeconomic strata in their analysis. However, studies that did include such strata observed that higher income groups had greater vaccine acceptance rates (Table S2). This is especially concerning in a country like India, which is classified as a low- and middle-income country. Even though our results are similar to those of previous studies from LMIC countries, the extent to which this phenomenon affects a country like India is much greater, as the proportion of those living in lower economic strata is higher. Although there are currently no studies to explain the reasons for the higher rates of vaccine refusal and hesitancy in this group, these rates are likely related to health beliefs and practices.

Female participants were found to be 1.48 times more likely to refuse the vaccine. Studies from LMICs observed that gender differences did not reveal consistent behavior in vaccine hesitancy and refusal. However, a larger number of studies concluded that males were more likely to be vaccinated (Table S2). This gender disparity was also seen in systematic reviews that included both HICs and LMICs and in those that included only LMICs [24,25,26,27]. This disparity may be due to various social and circumstantial influences such as rampant reports of women being more vulnerable to adverse vaccine effects [28] and reports of vaccines affecting the menstrual cycle [29] or current or planned pregnancy. Policymakers should seek to provide positive messages tailored specifically to female audiences in local languages. This gender disparity not only influences women, but may influence their children as well, to avoid receiving the vaccine.

Data on hesitancy to accept the COVID-19 vaccine vary between countries and are indicated in various systematic reviews conducted on the topic [25,26]. Vaccine hesitancy rates between countries were found to vary considerably between approximately 14% and 40%, and vaccine refusal varied between 3% and 33% [26]. In our research, 31% of the study population displayed vaccine hesitancy, which is slightly above the global average but in line with a systematic review on COVID-19 vaccine hesitancy carried out only among LIMCs, which was 38.2% [27]. The vaccine refusal rate was about 9%, which is significantly lower than the global average. The higher proportion of study participants expressing hesitancy rather than downright refusal toward the vaccine means that India has a unique opportunity to develop successful vaccine programs, as it is known that hesitant people may be more inclined to accept potential interventions [30]. During the same period, a smaller study in India with a smaller sample size of 280 observed a higher vaccine hesitancy rate of more than 40%. Several studies were also conducted on vaccine hesitancy and refusal rates in LMICs (Table S2). Many LMICs have lower resources for adequate public education and lower literacy rates among their population and hence may differ from HICs in their vaccine hesitancy and refusal rates. The sample sizes in these studies varied from 410 in Pakistan to 173,178 in Brazil. The refusal rates varied from 2.5% in Brazil to 71.6% in Jordan (Table S2). The vaccine hesitancy rates ranged from 5.7% in Malaysia to 31% in Turkey.

Current research on vaccine refusal and hesitancy highlights the concerns of the population regarding unforeseen future effects of the vaccine. This study showed that those afraid of the vaccine’s long-term safety were three times less likely to take the vaccine than those who were not afraid. Mistrust in the benefits of the vaccine, however, was found to be the strongest predictor of refusal and hesitancy to accept the vaccine.

Several global events concerning the vaccine have exacerbated these uncertainties. One of the COVID-19 vaccines developed by AstraZeneca reported several cases of blood clots, some leading to death, which resulted in many European countries pausing their vaccination campaigns [31]. Though concerns have been raised about the development of blood clots, there appear to be limited differences in reality [32]. A few countries such as Russia and the USA were accused of rushing the vaccine to market for general use with minimal data, with suspicions that vaccine rollouts were politically driven [33,34]. Such events, along with the growing trend of misinformation on social media and discussions on network television have made it difficult to predict if vaccination coverage will reach the levels required for herd immunity [35,36]. This misinformation, especially surrounding the Oxford AZ vaccine, may have increased hesitancy rates across countries. The extent of the misinformation surrounding effective treatments for COVID-19 propagated by social media is not new and was seen earlier for HCQ and Ivermectin in India and South Africa, respectively, despite concerns of side effects [37,38,39].

Our study, along with many others, has revealed identifiable subgroups that refuse or are hesitant to accept the vaccine [19,25,40,41,42,43,44]. Health policymakers should recognize these subgroups and tailor vaccine advisory campaigns to them.

“Fake news” has a large potential to influence community vaccine acceptance. The WHO designated fake news disseminated through digital media as an infodemic, while UNESCO called it a disinfodemic. The interrelationships between implicit learning, digital media, and fake news lead to negative epistemic post-digital inculcation [45]. The use of text and videos on social media such as Facebook, Twitter, and YouTube has led to situations that negatively impact COVID-19-related public health [46,47]. Thus, it is crucial to reach out to locally trusted sources and have them seek to establish a transparent and trusted line of communication with high-risk groups, as such sources are more effective in dealing with community-specific concerns and misconceptions [35]. There is also a clear need for campaigns to educate high-risk groups by explaining how vaccines are developed and when a vaccine is considered safe for usage. Print and electronic media can play an essential role in promoting vaccine acceptance, making appropriate engagement through such avenues indispensable. Other parameters such as the efficacy of vaccines, their dosage, and their limitations should also be made clear. These steps have worked well in previous infectious disease outbreaks and pandemics and could also apply to the COVID-19 pandemic [48].

Furthermore, this reality provides policymakers a unique opportunity to develop a trusting relationship with people, which will help further improve the outlook of the public on national vaccination programs in the country. This is the first study in India on the extent of vaccine refusal or hesitancy with a good sample size across major sociodemographic groups. The study evaluated not only those who are unwilling to accept but also the perception of subjects who were undecided about the vaccine.

However, our study has limitations. This is a cross-sectional study carried out at a single point in time. With the fast-paced changes in the pandemic, people’s vaccination intentions might change over time as new information becomes available. Furthermore, our outcome variable was a measure of future behavior rather than of actual or past behavior and thus may not be accurate. The data, moreover, were collected using an online survey, so there is a risk of inaccurate self-reporting. Even though large efforts were made to make the sample population as representative of the general population as possible, few participants were aged >65 or belonged to very high- or very low-income groups. Not all the participants completed the survey, and it is not clear how different the non-completion group was from the group that completed the survey.

## 5. Conclusions

In conclusion, almost one-third of the study population expressed hesitancy regarding vaccination. A generic information tool with a one-size-fits-all approach may not be sufficient to achieve high vaccine acceptance rates. There is a need for focused and targeted interventions for the specific vulnerable groups identified in our study based on their knowledge, attitudes, and practices. Since our hesitancy rates were nearly one-third those of the population surveyed, there is a need to develop vaccine education programs to help eliminate factors that contribute to vaccine hesitancy. This education, in turn, will help convert those who are unsure about the vaccine into acceptors.

There is also an urgent need for more detailed analysis, including qualitative interviews of different subgroups of subjects to further delineate the reasons for vaccine hesitancy or refusal, as India is a very diverse country with people of varying ethnicities and socioeconomic and cultural backgrounds.

## Figures and Tables

**Table 1:** Sociodemographic characteristics of the study population used to determine vaccine hesitancy, India, 2021 (*n* = 1582).

Variables	Number	Percentage
Gender		
Male	945	59.7%
Female	637	40.3%
Geographic quadrants		
North	427	26.4%
South	538	34.6%
West	259	16.4%
East	358	22.6%
Age (years)		
18–24	461	29.1%
25–44	622	39.3%
45–64	437	27.6%
65+	62	3.9%
Educational attainment		
Postgraduate education	431	27.2%
Undergraduate education	573	36.2%
Secondary school education	291	18.4%
Not willing to disclose	287	18.1%
Approximate family income		
≥199,862	151	9.5%
99,931–199,861	229	14.5%
74,756–99,930	346	21.9%
49,962–74,755	277	17.5%
29,973–49,961	64	4.0%
10,002–29,972	16	1.0%
≤10,001	83	5.2%
Not willing to disclose	416	26.3%
Living area		
Urban	1087	68.7%
Rural	495	31.3%
Knowledge of COVID-19 *		
Good knowledge	1211	76.5%
Poor knowledge	371	23.5%
Adherence to government COVID-19 guidelines		
Very much following	964	60.9%
Following less	618	39.1%
Have had COVID-19		
Have not had COVID-19	1017	64.3%
Have had COVID-19	565	35.7%
Coronavirus Anxiety Symptoms (CAS) **		
Ever ≥ 1 CAS symptom	201	12.7%
Never CAS symptoms	1381	87.3%

* Knowledge of COVID-19 was assessed using a separate six-question survey based on a questionnaire developed by Zhong et al. [15]. A total score of 1 to 3 was considered poor knowledge, and 4 to 6 was considered good knowledge. ** A CAS score of nine or more classified adults as meeting the threshold for Generalized Anxiety Disorder [13]. We categorized responses such that participants with one or more COVID-19 anxiety symptoms were compared to those who did not report any such symptoms. Responses to the question on compliance with government COVID-19 guidelines were assessed on a scale from 1 to 7, with 1 being “none at all” and 7 being “very much so.”

## Data Availability

All data generated or analyzed during this study are included in the published article and are available from the corresponding author upon reasonable request.

## Supplementary Materials

The following supporting information can be downloaded at: https://www.mdpi.com/2076-393X/10/7/1095/s1. Table S1: Strobe Statement for the study, Table S2: Studies conducted on the general population of other LMICs testing vaccine acceptance and related factors. References [49,50,51,52,53,54,55,56,57,58,59,60,61,62,63,64,65,66,67,68,69,70,71,72,73,74] are cited in the supplementary materials.
