# Supplementary material for: Willingness to Accept the COVID-19 Vaccine and Related Factors among Indian Adults: A Cross-Sectional Study
Source: Vaccines (Basel). 2022 Jul 8;10(7):1095. doi: 10.3390/vaccines10071095 (PMC9317945; doi:10.3390/vaccines10071095)
Supplement: Supplementary file 1 [file vaccines-10-01095-s001.zip › vaccines-1752152-supplementary.pdf]

vaccines

MDPI

Article

Supplementary Material: Willingness to Accept the COVID-19 Vaccine and Related Factors among Indian Adults: A Cross-Sectional Study

Ashwaghosha Parthasarathi [1], Rahul Krishna Puvvada [2], Malavika Shankar [3], Jayaraj Biligere Siddaiah [4], Koustav Ganguly [5], Swapna Upadhyay [5,*] and Padukudru Anand Mahesh [4,*]

[1] Allergy, Asthma and Chest Centre, Krishnamurthypuram, Mysore 570004, Karnataka, India; ashwa.partha@gmail.com
[2] Department of Physiology, Anatomy, and Microbiology, College of Science Health and Engineering, La Trobe University, Melbourne Victoria 3086, Australia; 20173174@students.latrobe.edu.au
[3] Department of Internal Medicine, Interfaith Medical Center, New York, NY 11213, USA; mshankar@interfaithmedical.com
[4] Department of Pulmonology, JSS Medical College, JSS Academy of Higher Education & Research, Mysore 570015, Karnataka, India; bsjayaraj@jssuni.edu.in
[5] Unit of Integrative Toxicology, Institute of Environmental Medicine (IMM), Karolinska Institutet, 17177 Stockholm, Sweden; koustav.ganguly@ki.se
* Correspondence: swapna.upadhyay@ki.se (S.U.); pamahesh@jssuni.edu.in (P.A.M.)

Table S1. STROBE Statement—checklist of items that should be included in reports of observational studies.

	Item No.	Recommendation	Page No.
**Title and abstract**	1	(*a*) Indicate the study's design with a commonly used term in the title or the abstract	2
		(*b*) Provide in the abstract an informative and balanced summary of what was done and what was found	2
Background/rationale	2	Explain the scientific background and rationale for the investigation being reported	3–4
Objectives	3	State specific objectives, including any prespecified hypotheses	4
Study design	4	Present key elements of study design early in the paper	4
Setting	5	Describe the setting, locations, and relevant dates, including periods of recruitment, exposure, follow-up, and data collection	4–5
Participants	6	a) ***Cohort study***—Give the eligibility criteria, and the sources and methods of selection of participants. Describe methods of follow-up<br>b) ***Case-control study***—Give the eligibility criteria, and the sources and methods of case ascertainment and control selection. Give the rationale for the choice of cases and controls<br>c) ***Cross-sectional study***—Give the eligibility criteria, and the sources and methods of selection of participants	4–5
		d) ***Cohort study***—For matched studies, give matching criteria and number of exposed and unexposed<br>*e)* ***Case-control study***—For matched studies, give matching criteria and the number of controls per case	NA
Variables	7	Clearly define all outcomes, exposures, predictors, potential confounders, and effect modifiers. Give diagnostic criteria, if applicable	5–6
Data sources/ measurement	8 *	For each variable of interest, give sources of data and details of methods of assessment (measurement). Describe comparability of assessment methods if there is more than one group	*6*
Bias	9	Describe any efforts to address potential sources of bias	NA
Study size	10	Explain how the study size was arrived at	4

Quantitative variables	11	Explain how quantitative variables were handled in the analyses. If applicable, describe which groupings were chosen and why	6
Statistical methods	12	(*a*) Describe all statistical methods, including those used to control for confounding	6
		(*b*) Describe any methods used to examine subgroups and interactions	NA
		(*c*) Explain how missing data were addressed	6
		(*d*) *Cohort study*—If applicable, explain how loss to follow-up was addressed	
		Case-control study—If applicable, explain how matching of cases and controls was addressed	NA
		Cross-sectional study—If applicable, describe analytical methods taking account of sampling strategy	
		(*e*) Describe any sensitivity analyses	NA
Participants	13 *	(a) Report numbers of individuals at each stage of study—eg numbers potentially eligible, examined for eligibility, confirmed eligible, included in the study, completing follow-up, and analysed	7
		(b) Give reasons for non-participation at each stage	NA
		(c) Consider use of a flow diagram	NA
Descriptive data	14 *	(a) Give characteristics of study participants (eg demographic, clinical, social) and information on exposures and potential confounders	7
		(b) Indicate number of participants with missing data for each variable of interest	7
		(c) *Cohort study*—Summarise follow-up time (eg, average and total amount)	NA
Outcome data	15 *	*Cohort study*—Report numbers of outcome events or summary measures over time	*NA*
		Case-control study—Report numbers in each exposure category, or summary measures of exposure	*NA*
		Cross-sectional study—Report numbers of outcome events or summary measures	*7–9*
Main results	16	(*a*) Give unadjusted estimates and, if applicable, confounder-adjusted estimates and their precision (eg, 95% confidence interval). Make clear which confounders were adjusted for and why they were included	8–9
		(*b*) Report category boundaries when continuous variables were categorized	7
		(*c*) If relevant, consider translating estimates of relative risk into absolute risk for a meaningful time period	NA
Other analyses	17	Report other analyses done—eg analyses of subgroups and interactions, and sensitivity analyses	NA
Key results	18	Summarise key results with reference to study objectives	10–13
Limitations	19	Discuss limitations of the study, taking into account sources of potential bias or imprecision. Discuss both direction and magnitude of any potential bias	13
Interpretation	20	Give a cautious overall interpretation of results considering objectives, limitations, multiplicity of analyses, results from similar studies, and other relevant evidence	13–14
Generalisability	21	Discuss the generalisability (external validity) of the study results	13
Funding	22	Give the source of funding and the role of the funders for the present study and, if applicable, for the original study on which the present article is based	15

*Give information separately for exposed and unexposed groups. NA: Not applicable

Table S2. Studies done on the general population of other LMICs testing vaccine acceptance and related factors.

Author	Country	Sample size (N), Age range or M ± SD	Comparison information	Intention results	Age	Gender	Education	Income
M Abedin (2021)[1]	Bangladesh	3646, <15 to 65 years) / 37.4 ± 13.9	Vaccine acceptance Vs Refusal Vs Hesitancy	Yes: 74.5% No: 8.5% Unsure: 17%	Not significant	Not significant	Not significant	-
Acheampong T (2021) [2]	Ghana	2345, 15 to > 55 years	Vaccine acceptance Vs Refusal Vs Hesitancy	Yes: 51% No: 21% Unsure: 28%	Age above 55 years are more likely to vaccinate	Males more likely to vaccinate (56%)	Higher educational qualification more likely to vaccinate (59%)	-
Adebisi YA (2021) [3]	Nigeria	517, 16 to ≥61 years	Vaccine acceptance Vs Refusal	Yes: 74.5% No: 24%	Adults (16 to 30 years) are more likely to vaccinate	Males more likely to vaccinate	Higher educational qualification more likely to vaccinate	-
Ahmed MAM (2021) [4]	Somalia	4543, 23.5 ± 6.4 years	Vaccine acceptance Vs Refusal	Yes: 76.7% No: 23.3%	Not significant	Females more likely to vaccinate	Not significant	Not significant
Ahmed TF (2021) [5]	Pakistan	655, 18 to ≥60 years	Vaccine acceptance Vs Refusal Vs Hesitancy	Yes: 62% No: 15.6% Unsure: 22.4%	Age above 50 years are more likely to vaccinate	Not significant	Not significant	High income participants are willing to take vaccine.

Akiful Haque MM (2021) [6]	Bangladesh	7357, <30 to > 50 years	Vaccine acceptance Vs Refusal Vs Hesitancy	Yes: 65.05%	Age above 30 years are more likely to vaccinate	Not significant	Higher educational qualification more likely to vaccinate	High income participants are willing to take vaccine.
WA Al-Qerem (2021)[7]	Jordan	1144, 18 to 60 years	Vaccine acceptance Vs Refusal Vs Hesitancy	Yes: 36.8% No: 36.8% Unsure: 26.4%	Not significant	Females are unwilling to take vaccine.	University students are willing to take vaccine.	-
AJ Udoakang (2022)[8]	West Africa	1106, ≤20 to ≥41 years / 32.14 ± 8.35	Vaccine acceptance Vs Refusal Vs Hesitancy	Yes: 75.2% No: 10.4% Unsure: 14.4%	Age above 41 years are willing to take vaccine (85.2%)	Males are willing to take vaccine (75.8%)	Higher educational qualification are willing to take vaccine (77.8%)	-
Arshad MS (2021) [9]	Pakistan	2158, 18 to >50 years	Vaccine acceptance Vs Refusal Vs Hesitancy	Yes: 48.2% No: 25.8% Unsure: 26%	-	-	-	-
Bongomin F (2021) [10]	Uganda	317, 51.5 ± 14.1 years	Vaccine acceptance Vs Refusal	Yes: 70.1% No: 29.9%	-	Males are willing to take vaccine	Not significant	-
Bono SA (2021) [11]	Bangladesh	230, 18 to ≥60 years 28.62 ± 6.53	Vaccine acceptance Vs Hesitancy	Yes: 89.57	Young people (18–39 years) are willing to take vaccine	Males are willing to take vaccine	Higher educational qualification are willing to take vaccine	High income participants are willing to take vaccine
	DR Congo	219, 18 to ≥60 years, 35.20 ± 8.96	Vaccine acceptance VS hesitancy	Yes: 59.36	Young people (18–39 years)	Males are willing to take vaccine	Higher educational	High income participants are

					are willing to take vaccine		qualification are willing to take vaccine	willing to take vaccine
	Benin	159, 18 to ≥60 years, 28.50±10.24	Vaccine acceptance VS hesitancy	Yes: 48.43	Young people (18–39 years) are willing to take vaccine	Males are willing to take vaccine	Higher educational qualification are willing to take vaccine	High income participants are willing to take vaccine
	Uganda	107, 18 to ≥60 years, 33.79 ± 8.84	Vaccine acceptance VS hesitancy	Yes: 88.79	Young people (18–39 years) are willing to take vaccine	Males are willing to take vaccine	Higher educational qualification are willing to take vaccine	High income participants are willing to take vaccine
	Malawi	81, 18 to ≥60 years, 37.80 ± 8.63	Vaccine acceptance VS hesitancy	Yes: 61.73	Young people (18–39 years) are willing to take vaccine	Males are willing to take vaccine	Higher educational qualification are willing to take vaccine	High income participants are willing to take vaccine
	Mali	55, 18 to ≥60 years, 37.44 ± 8.99	Vaccine acceptance VS hesitancy	Yes: 74.55	Young people (18–39 years) are willing to take vaccine	Males are willing to take vaccine	Higher educational qualification are willing to take vaccine	High income participants are willing to take vaccine
Chaudhary FA (2021) [12]	Pakistan	410, 18 to > 52 years /	Vaccine acceptance Vs Refusal	Yes: 52.7% No: 47.3%	Adults (18–30yrs) are willing to take vaccine	Females are willing to take vaccine (60.2%)	Higher educational qualification are willing to take vaccine (51.9%)	Higher income group are willing to take vaccine (30.6%)
DCBC Moore (2021) [13]	Brazil	173,178, 18 to ≥ 75 years	Vaccine acceptance Vs Refusal Vs Hesitancy	Yes: 89.5% No:2.5% Unsure: 8%	Adults (18–39years) are willing to	Females are willing to take vaccine (91.2%)	Higher educational qualification are	High income groups are willing to take vaccine (90.3%)

					take vaccine (91.3%)		willing to take vaccine (90.1%)	
D Damba-darjaa (2021) [14]	Mongolia	2875, 18 to ≥ 50 years	Vaccine acceptance Vs Refusal Vs Hesitancy	Yes: 68.3% No: 7.1% Unsure: 24.5%	Age above 50 years are willing to take vaccine (80.7%)	Females are willing to take vaccine (68.4%)	Higher educational qualification are willing to take vaccine (60%)	-
Harapan (2020) [15]	Indonesia	1359, <20 to >51 years	Vaccine acceptance Vs Refusal	Yes: 93.3% No: 6.7%	Not significant	Females are willing to take vaccine.	Not significant	Not significant
J Amo-Adjei (2022) [16]	Ghana	415, <20 to ≥60 years	Vaccine acceptance Vs Refusal Vs Hesitancy	Yes: 70% No: 20% Unsure: 9.9%	Age above 50 years are willing to take vaccine (87.5%)	Females are willing to take vaccine (73.5%)	Higher educational qualification are willing to take vaccine (73.4%)	-
LG Shareef (2022) [17]	Iraq	1221, 18 to >65 years	Vaccine acceptance Vs Refusal	Yes: 56.2% No: 43.8%	Adults (18–29yrs) are willing to take vaccine	Males are willing to take vaccine (29%)	Higher educational qualification are willing to take vaccine	-
LB. Tlale (2022) [18]	Botswana	5300, 18 to >65 years	Vaccine acceptance Vs Refusal	Yes: 73.4% No: 31.3%	Not significant	Males are willing to take vaccine.	Participants with primary educational qualification are willing to take vaccine.	-
M Bou Hamdan (2021) [19]	Lebanon	800, ≤20 to 40 years / 21 ± 0.14 years	Vaccine acceptance Vs Refusal Vs Hesitancy	Yes: 87% No: 3%	Adults (21–30 years) are willing to	Males are willing to	Higher educational qualification are	-

				Unsure: 10%	take vaccine (88%)	take vaccine (88%)	willing to take vaccine (88.5%)	
P Gaur (2021) [20]	India	280, 47 ± 13 years	Vaccine acceptance Vs Refusal	Yes: 57.5% No: 42.5%	Age above 45 years are willing to take vaccine.	-	Graduates are willing to take vaccine (69%)	-
GD Salali (2020) [21]	Turkey	3936	Vaccine acceptance Vs Refusal Vs Hesitancy	Yes: 66% No: 3% Unsure: 31%	-	Males are willing to take vaccine.	Higher education unwilling to take vaccine.	-
M Sallam (2020) [22]	Jordan	2173, 16 to > 40 years	Vaccine acceptance Vs Refusal	Yes: 28.4% No: 71.6%	-	Males are willing to take vaccine	Higher educational qualification are willing to take vaccine	-
SAR Syed Alwi (2021) [23]	Malaysia	1411, 18 to > 60 years	Vaccine acceptance Vs Refusal Vs Hesitancy	Yes: 83.3% Unsure: 16.7%	Adults (18–29years) are willing to take vaccine (88.3%)	Females are willing to take vaccine (83.5%)	Higher educational qualification are willing to take vaccine (83%)	High income groups are willing to take vaccine (81%)
Solis Arce (2021) [24]	Pakistan 1	1633	Vaccine acceptance Vs Refusal Vs Hesitancy	Yes: 76.12	Adults (<25 years) are willing to take vaccine	Males are willing to take vaccine	Not significant	-
	Pakistan 2	1492	Vaccine acceptance Vs Refusal Vs Hesitancy	Yes: 66.49	Adults (<25 years) are willing to take vaccine	Males are willing to take vaccine	Not significant	-

	Rwanda	1355	Vaccine acceptance Vs Refusal Vs Hesitancy	Yes: 84.87	Adults (<25 years) are willing to take vaccine	Males are willing to take vaccine	Not significant	-
	Sierra Leone 1	1070	Vaccine acceptance Vs Refusal Vs Hesitancy	Yes: 78.04	Adults (<25 years) are willing to take vaccine	Males are willing to take vaccine	Not significant	-
	Sierra Leone 2	2110	Vaccine acceptance Vs Refusal Vs Hesitancy	Yes: 87.91	Adults (<25 years) are willing to take vaccine	Males are willing to take vaccine	Not significant	-
	Uganda 1	3362	Vaccine acceptance Vs Refusal Vs Hesitancy	Yes: 85.81	Adults (<25 years) are willing to take vaccine	Males are willing to take vaccine	Not significant	-
	Uganda 2	1366	Vaccine acceptance Vs Refusal Vs Hesitancy	Yes: 76.50	Adults (<25 years) are willing to take vaccine	Males are willing to take vaccine	Not significant	-
LP Wong (2020) [25]	Malaysia	1159, 18 to > 50 years	Vaccine acceptance Vs Refusal	Yes: 94.3% No: 5.7%	Adults (18–30 years) are willing to take vaccine	Males are willing to take vaccine.	-	-
Qunaibi EA (2021) [26]	Algeria	2706	Vaccine acceptance Vs Refusal Vs Hesitancy	Yes: 3.62% No: 61.9% Unsure: 18.8%	Adults (18–29 years) are willing to take vaccine	Males are willing to take vaccine.	Higher educational qualification are	-

						willing to take vaccine	
Egypt	5339	Vaccine acceptance Vs Refusal Vs Hesitancy	Yes: 8% No: 36.5 Unsure: 21.8%	Adults (18–29 years) are willing to take vaccine	Males are willing to take vaccine.	Higher educational qualification are willing to take vaccine	-
Mauritania	99	Vaccine acceptance Vs Refusal Vs Hesitancy	Yes: 8% No: 39.4% Unsure: 32.3%	Adults (18–29 years) are willing to take vaccine	Males are willing to take vaccine.	Higher educational qualification are willing to take vaccine	-
Morocco	3775	Vaccine acceptance Vs Refusal Vs Hesitancy	Yes: 7.9% No: 46.4% Unsure: 25.5%	Adults (18–29 years) are willing to take vaccine	Males are willing to take vaccine.	Higher educational qualification are willing to take vaccine	-
Sudan	313	Vaccine acceptance Vs Refusal Vs Hesitancy	Yes: 15.3% No: 43.5% Unsure: 19.2%	Adults (18–29 years) are willing to take vaccine	Males are willing to take vaccine.	Higher educational qualification are willing to take vaccine	-
Syria	1232	Vaccine acceptance Vs Refusal Vs Hesitancy	Yes: 10.7% No: 43.5% Unsure: 19.2%	Adults (18–29 years) are willing to take vaccine	Males are willing to take vaccine.	Higher educational qualification are willing to take vaccine	-

	Tunisia	665	Vaccine acceptance Vs Refusal Vs Hesitancy	Yes: 6.5% No: 53.8% Unsure: 18%	Adults (18–29 years) are willing to take vaccine	Males are willing to take vaccine.	Higher educational qualification are willing to take vaccine	-
	Yemen	226	Vaccine acceptance Vs Refusal Vs Hesitancy	Yes: 9.3% No: 54.4% Unsure: 14.6%	Adults (18–29 years) are willing to take vaccine	Males are willing to take vaccine.	Higher educational qualification are willing to take vaccine	-

References:

1. Abedin, M.; Islam, M.A.; Rahman, F.N.; Reza, H.M.; Hossain, M.Z.; Hossain, M.A.; Arefin, A.; Hossain, A. Willingness to vaccinate against COVID-19 among Bangladeshi adults: Understanding the strategies to optimize vaccination coverage. *PLoS One* **2021**, 16(4): e0250495. https://doi.org/10.1371/journal.pone.0250495
2. Acheampong, T.; Akorsikumah, E.A.; Osae-Kwapong, J.; Khalid, M.; Appiah, A.; Amuasi, J.H. Examining Vaccine Hesitancy in Sub-Saharan Africa: A Survey of the Knowledge and Attitudes among Adults to Receive COVID-19 Vaccines in Ghana. *Vaccines* **2021**, *9*, 814, doi:10.3390/vaccines9080814.
3. Adebisi, Y.A.; Alaran, A.J.; Bolarinwa, O.A.; Akande-Sholabi, W.; Lucero-Prisno, D.E. When it is available, will we take it? Social media users' perception of hypothetical COVID-19 vaccine in Nigeria. *Pan Afr Med J* **2021**, *38*, 230–230, doi:10.11604/pamj.2021.38.230.27325.
4. Ahmed, M.A.M.; Colebunders, R.; Gele, A.A.; Farah, A.A.; Osman, S.; Guled, I.A.; Abdullahi, A.A.; Hussein, A.M.; Ali, A.M.; Siewe Fodjo, J.N. COVID-19 Vaccine Acceptability and Adherence to Preventive Measures in Somalia: Results of an Online Survey. *Vaccines* **2021**, *9*, doi:10.3390/vaccines9060543.
5. Ahmed, T.F.; Ahmed, A.; Ahmed, S.; Ahmed, H.U. Understanding COVID-19 vaccine acceptance in Pakistan: an echo of previous immunizations or prospect of change? *Expert Rev. of Vaccines* **2021**, *20*, 1185–1193, doi:10.1080/14760584.2021.1964963.
6. Akiful Haque, M.M.; Rahman, M.L.; Hossian, M.; Matin, K.F.; Nabi, M.H.; Saha, S.; Hasan, M.; Manna, R.M.; Barsha, S.Y.; Hasan, S.M.R., et al. Acceptance of COVID-19 vaccine and its determinants: evidence from a large sample study in Bangladesh. *Heliyon* **2021**, *7*, e07376-e07376, doi:10.1016/j.heliyon.2021.e07376.
7. Al-Qerem, W.A.; Jarab, A.S. COVID-19 Vaccination Acceptance and Its Associated Factors Among a Middle Eastern Population. *Front Public Health* **2021**, *9*, 632914–632914, doi:10.3389/fpubh.2021.632914.
8. Udoakang, A.J.; Djomkam Zune, A.L.; Tapela, K.; Owoicho, O.; Fagbohun, I.K.; Anyigba, C.A.; Lowe, M.; Nganyewo, N.N.; Keneme, B.; Olisaka, F.N., et al. Knowledge, attitude and perception of West Africans towards COVID-19: a survey to inform public health intervention. *BMC Public Health* **2022**, *22*, 445, doi:10.1186/s12889-022-12814-9.
9. Arshad, M.S.; Hussain, I.; Mahmood, T.; Hayat, K.; Majeed, A.; Imran, I.; Saeed, H.; Iqbal, M.O.; Uzair, M.; Rehman, A.U., et al. A National Survey to Assess the COVID-19 Vaccine-Related Conspiracy Beliefs, Acceptability, Preference, and Willingness to Pay among the General Population of Pakistan. *Vaccines* **2021**, *9*, 720, doi:10.3390/vaccines9070720.
10. Bongomin, F.; Olum, R.; Andia-Biraro, I.; Nakwagala, F.N.; Hassan, K.H.; Nassozi, D.R.; Kaddumukasa, M.; Byakika-Kibwika, P.; Kiguli, S.; Kirenga, B.J. COVID-19 vaccine acceptance among high-risk populations in Uganda. *Therapeutic Advances in Infectious Disease* **2021**, *8*, 20499361211024376, doi:10.1177/20499361211024376.
11. Bono, S.A.; Faria de Moura Villela, E.; Siau, C.S.; Chen, W.S.; Pengpid, S.; Hasan, M.T.; Sessou, P.; Ditekemena, J.D.; Amodan, B.O.; Hosseinipour, M.C., et al. Factors Affecting COVID-19 Vaccine Acceptance: An International Survey among Low- and Middle-Income Countries. *Vaccines (Basel)* **2021**, *9*, doi:10.3390/vaccines9050515.
12. Chaudhary, F.A.; Ahmad, B.; Khalid, M.D.; Fazal, A.; Javaid, M.M.; Butt, D.Q. Factors influencing COVID-19 vaccine hesitancy and acceptance among the Pakistani population. *Human Vaccines Immunotherapeutics* **2021**, *17*, 3365–3370, doi:10.1080/21645515.2021.1944743.
13. Moore, D.C.B.C.; Nehab, M.F.; Camacho, K.G.; Reis, A.T.; Junqueira-Marinho, M.d.F.; Abramov, D.M.; Azevedo, Z.M.A.d.; Menezes, L.A.d.; Salú, M.d.S.; Figueiredo, C.E.d.S., et al. Low COVID-19 vaccine hesitancy in Brazil. *Vaccine* **2021**, *39*, 6262–6268, doi:https://doi.org/10.1016/j.vaccine.2021.09.013.
14. Dambadarjaa, D.; Altankhuyag, G.-E.; Chandaga, U.; Khuyag, S.-O.; Batkhorol, B.; Khaidav, N.; Dulamsuren, O.; Gombodorj, N.; Dorjsuren, A.; Singh, P., et al. Factors Associated with COVID-19 Vaccine Hesitancy in Mongolia: A Web-Based Cross-Sectional Survey. *Int. J. of Environ. Res. Public Health* **2021**, *18*, doi:10.3390/ijerph182412903.

15. Harapan, H.; Wagner, A.L.; Yufika, A.; Winardi, W.; Anwar, S.; Gan, A.K.; Setiawan, A.M.; Rajamoorthy, Y.; Sofyan, H.; Mudatsir, M. Acceptance of a COVID-19 Vaccine in Southeast Asia: A Cross-Sectional Study in Indonesia. *Front Public Health* **2020**, *8*, 381–381, doi:10.3389/fpubh.2020.00381.
16. Amo-Adjei, J.; Nurzhynska, A.; Essuman, R.; Lohiniva, A.-L. Trust and willingness towards COVID-19 vaccine uptake: a mixed-method study in Ghana, 2021. *Archives of Public Health* **2022**, *80*, 64, doi:10.1186/s13690-022-00827-0.
17. Shareef, L.G.; Fawzi Al-Hussainy, A.; Majeed Hameed, S. COVID-19 vaccination hesitancy among Iraqi general population between beliefs and barriers: An observational study. *F1000Res* **2022**, *11*, 334, doi:10.12688/f1000research.110545.2.
18. Tlale, L.B.; Gabaitiri, L.; Totolo, L.K.; Smith, G.; Puswane-Katse, O.; Ramonna, E.; Mothowaeng, B.; Tlhakanelo, J.; Masupe, T.; Rankgoane-Pono, G., et al. Acceptance rate and risk perception towards the COVID-19 vaccine in Botswana. *PLoS One* **2022**, *17*, e0263375, doi:10.1371/journal.pone.0263375.
19. Bou Hamdan, M.; Singh, S.; Polavarapu, M.; Jordan, T.R.; Melhem, N.M. COVID-19 vaccine hesitancy among university students in Lebanon. *Epidemiol. Infect* **2021**, *149*, e242, doi:10.1017/S0950268821002314.
20. Gaur, P.; Agrawat, H.; Shukla, A. COVID-19 vaccine hesitancy in patients with systemic autoimmune rheumatic disease: an interview-based survey. *Rheumatol Int* **2021**, *41*, 1601–1605, doi:10.1007/s00296-021-04938-9.
21. Salali, G.D.; Uysal, M.S. COVID-19 vaccine hesitancy is associated with beliefs on the origin of the novel coronavirus in the UK and Turkey. *Psychol. Med.* **2020**, 10.1017/S0033291720004067, 1–3, doi:10.1017/S0033291720004067.
22. Sallam, M.; Dababseh, D.; Eid, H.; Al-Mahzoum, K.; Al-Haidar, A.; Taim, D.; Yaseen, A.; Ababneh, N.A.; Bakri, F.G.; Mahafzah, A. High Rates of COVID-19 Vaccine Hesitancy and Its Association with Conspiracy Beliefs: A Study in Jordan and Kuwait among Other Arab Countries. *Vaccines* **2021**, *9*, 42, doi:10.3390/vaccines9010042.
23. Syed Alwi, S.A.R.; Rafidah, E.; Zurraini, A.; Juslina, O.; Brohi, I.B.; Lukas, S. A survey on COVID-19 vaccine acceptance and concern among Malaysians. *BMC Public Health* **2021**, *21*, 1129, doi:10.1186/s12889-021-11071-6.
24. Solís Arce, J.S.; Warren, S.S.; Meriggi, N.F.; Scacco, A.; McMurry, N.; Voors, M.; Syunyaev, G.; Malik, A.A.; Aboutajdine, S.; Adeojo, O., et al. COVID-19 vaccine acceptance and hesitancy in low- and middle-income countries. *Nature Medicine* **2021**, *27*, 1385–1394, doi:10.1038/s41591-021-01454-y.
25. Wong, L.P.; Alias, H.; Wong, P.-F.; Lee, H.Y.; AbuBakar, S. The use of the health belief model to assess predictors of intent to receive the COVID-19 vaccine and willingness to pay. *Hum. vaccines Immunother.* **2020**, *16*, 2204–2214, doi:10.1080/21645515.2020.1790279.
26. Qunaibi, E.A.; Helmy, M.; Basheti, I.; Sultan, I. A high rate of COVID-19 vaccine hesitancy in a large-scale survey on Arabs. *Elife* **2021**, *10*, e68038, doi:10.7554/eLife.68038.
